# The influence of boreal summer intra-seasonal oscillations on Indo-western Pacific Ocean surface waves

**DOI:** 10.1038/s41598-020-69496-9

**Published:** 2020-07-28

**Authors:** G. Srinivas, P. G. Remya, S. Malavika, T. M. Balakrishnan Nair

**Affiliations:** 1 Indian National Centre for Ocean Information Services (INCOIS), Ministry of Earth Sciences, Government of India, Hyderabad, 500090 India; 2 Cochin University of Science and Technology (CUSAT), Cochin, 682022 India

**Keywords:** Climate sciences, Ocean sciences

## Abstract

The present study examines the influence of Boreal Summer Intra-Seasonal Oscillation (BSISO) on Tropical Indian Ocean surface waves using the latest version of ECMWF reanalysis (ERA5) during summer monsoon months June through August (JJA). BSISO is a distinct mode of ISO during JJA having a northward and eastward movement from the equatorial Indian Ocean to the western Pacific Ocean. Composite analysis of anomalies of significant wave heights (SWH), wind sea, swell, and mean wave period for 8 phases of BSISO has been carried out to understand its influence. SWH anomalies in response to BSISO’s are phase-dependent. Negative SWH anomalies are noticed with strong northward and weak eastward propagation during the phases 1–3 in response to the easterly wind anomalies over the north Indian Ocean (NIO). During phases 5–7, high positive SWH anomalies (~ 0.5 m) in response to the westerly wind anomalies with northward and weak eastward propagation over NIO. Phases 4 and 8 behave like transition phases. In addition, enhanced (suppressed) SWH anomalies (~ 0.5 m) are seen during the active (break) spells of BSISO over NIO. Over the southern tip of India, negative (positive) SWH anomalies prevail during the active (break) conditions. This study clearly suggests that the wave forecast advisories during intra-seasonal time scales would be more useful for offshore and coastal activities during the summer monsoon.

## Introduction

The seasonal variations in the tropical Intra Seasonal Oscillation (ISO) are usually represented with two distinct modes namely Madden Julian Oscillation (MJO) and Boreal Summer Intra Seasonal Oscillation (BSISO). BSISO dominates during boreal summer (June–October) and MJO dominates during boreal winter (December–April)^1–3^. BSISO consist of alternating episodes of active and suppressed convection moving northward with 30–60 day mode in the Indian Ocean (IO) and westward propagating 10–20 day mode in the northwestern tropical Pacific Ocean, both together referred as BSISO and it shows more complexity in nature as compared to MJO^4–7^. It has a large variability in off-equatorial monsoon trough regions and is also considered as one of the prominent sources of short-term climate variability in the Asian summer monsoon^8^. The most important feature of BSISO is the meridional propagation of clouds and convection from about 5°S to 25°N over the South Asian monsoon region^2,4,9^. The large-scale air–sea interaction can only modulate the amplitude, frequency domain, and northward propagation characteristics of BSISO^10–13^. Also, the potential influence of BSISO on the seasonal mean monsoon is well addressed in many previous studies^14–16^. All these studies explain that the active (break) phase of BSISO is strengthening (weakening) the seasonal mean and playing a critical role in shaping the seasonal mean monsoon. Additionally, many researchers studied its influence on large-scale circulation and moisture processes, rainfall variability and flooding in the Asian monsoon region, temperature variability, and extreme heat wave events, tropical cyclone formation etc.^17–19^.

The surface wind climate in the north IO is under the direct influence of the summer monsoon. The monsoon wind pattern in the NIO especially over the Arabian Sea (AS) is complex in many ways and also with the presence of two low-level jets, the Findlater (or Somali) jet, and the Oman coastal jet. These complex monsoon wind patterns create a seasonal variability and influence the wave climate over the regions. The characteristics of the wind generated surface waves changes drastically over the NIO during the summer monsoon months (June–September) compared to the rest of the year^20–23^. The occurrence of monsoon waves higher than 4 m once and/or more times every year in the eastern AS has become common in recent years^24,25^. Importantly, monsoon waves have a considerable impact on the coastal and offshore infrastructures, navigation and etc. over the NIO and play a crucial role in the coastal dynamics and beach erosion. Monsoon waves are identified as an important driver of coastal erosion^26^ and cause extensive beach erosion and flooding events along the west coast of India^27^.

Ocean surface waves display variability from the intra-seasonal to interannual timescales^28^. In the interannual timescales, Indian Ocean Dipole (IOD), El Nino southern oscillation (ENSO), Southern Annular Mode (SAM) are the potential drivers for the wave variability over the Tropical Indian Ocean (TIO)^29–31^. The significant wave anomalies over the tropical north Atlantic and Pacific during the winter season and also the extra tropical wave signals with the occurrence of low and high wave conditions are in connection to the MJO^32^. However, the wave activity in the tropical IO is mainly modulated by the monsoon driven winds and BSISO which is the leading mode variability in the intra-seasonal scale during the boreal summer season. Hence, understanding the impact of BSISO mode on TIO surface wave heights during the boreal summer season (June–August) is crucial for the improvement of the wave forecast system which is beneficial for many marine applications and coastal communities. In the present study, we investigated the influence of BSISO on wind waves using ERA5 reanalysis data during JJA for the period (1979–2017).

## Data and methods

We have used the daily means of wave variables significant height of combined wind waves and swell (SWH), significant height of wind waves (Hsw), significant height of total swell (Hss), mean wave period (MWP) and surface winds (Ws) from the latest version of the European Centre for Medium-Range Weather Forecasts (ECMWF) reanalysis (ERA5) with the horizontal resolution 0.5° × 0.5° during the period 1979–2017^33^. By definition, the significant wave height, SWH, is defined as, $SWH=4\sqrt{{m}_{0}}$. The mean period Tm − 1 is based on the moment of order − 1, that is ${T}_{m-1}=\frac{{m}_{-1}}{{m}_{0}}$. The normalized energy flux is also used from ERA5, which is a normalized vertical flux of energy transfer from the wind into the ocean waves. A positive flux implies a flux into the waves. The energy flux has units of W/m^2^, and this is normalized by being divided by the product of air density and the cube of the friction velocity. The wave energy flux based on the wave power spectrum is defined as follows, $P_{w} = \mathop \smallint \limits_{0}^{2\pi } \mathop \smallint \limits_{0}^{\infty } Cg\left( {f,h} \right)S\left( {f,\theta } \right)dfd\theta$, where ρ, g, f, h, θ, Cg(f,h), and S(f,θ) are the seawater density, acceleration due to gravity, wave frequency, water depth, wave direction, group velocity, and spectral energy density, respectively. ERA5 combines model data with observations across the world to get a complete and consistent global dataset by using the laws of physics and data assimilation techniques. It provides hourly estimates of a large number of atmospheric, land and oceanic climate variables. The validation of ERA5 SWH with NIO buoy wave data shows a good comparison with low bias (− 0.03 to 0.07 m), low RMSE (0.2–0.27 m) and high correlation, R (0.94–0.98). The mean wave period also agrees well with the observation in the NIO [bias (− 0.23 to 0.32 s), RMSE (0.71–1 s), R (0.7–0.88)]. Few recent studies also showed the reliability of ERA5 wave data in the NIO by comparing it with in-situ and altimeter data^34, 35^. The daily outgoing long wave radiation (OLR) is taken from the National Centre for Environmental Prediction (NCEP) National Center for Atmospheric Research (NCAR) Reanalysis available with the horizontal resolution of 2.5° × 2.5°^36^.

The extended empirical orthogonal function (EEOF) analysis on daily OLR data has been used to extract the intra-seasonal scale leading mode BSISO (June through August) for the period 1979–2017^37^. Intra-seasonal anomalies has been extracted by applying the Lanczos band-pass filter^38^ with cut-off periods 25 and 90 days on the daily OLR data before performing the EEOF analysis. Composite analysis has been carried out on the life cycle of BSISO mode consists of 8 phases from P1 to P8.

## Results and discussions

**Figure 1:**
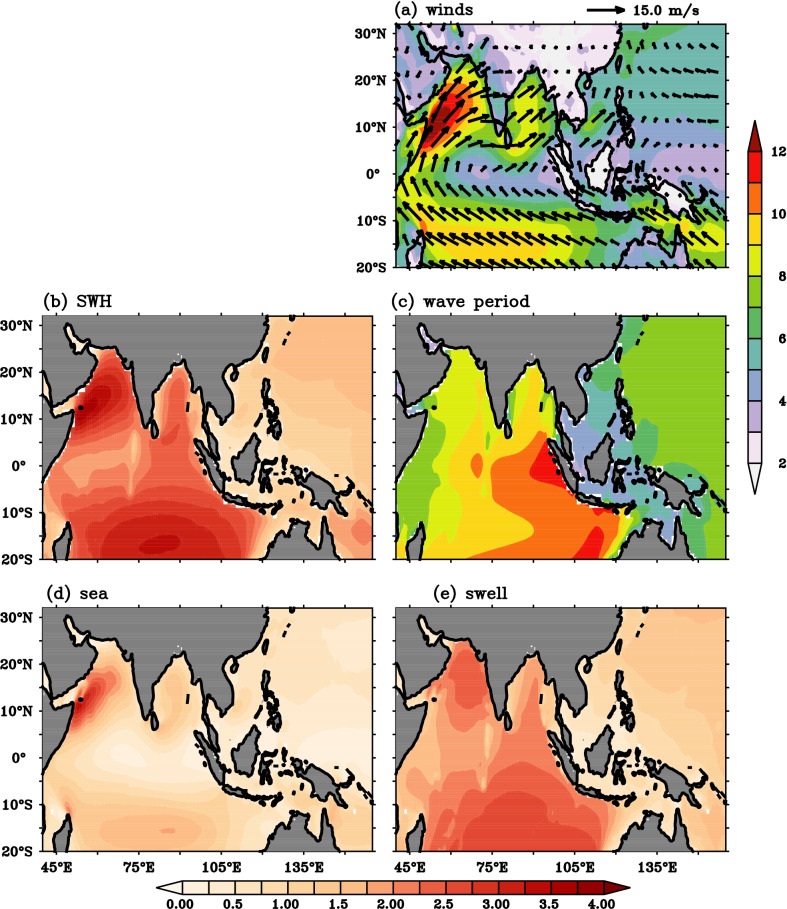
JJA Climatology of (**a**) surface winds (shaded and vectors; m/s), (**b**) significant wave height (m), (**c**) mean wave period (s), (**d**) significant wave height wind sea (m), and (**e**) significant swell wave height (m). This figure is generated using PyFerret software (https://ferret.pmel.noaa.gov/Ferret).

Climatological mean field of surface wind speed (Ws),significant wave height with combined sea and swells (SWH), significant height of wind sea (Hsw) , significant height total swell (Hss) and mean wave period (MWP) over tropical Indian Ocean (TIO) shown in Fig. 1 to sets the background for understanding the BSISO induced wave anomalies during JJA. Strong southwesterly winds with the speed more than 12 m/s (Fig. 1a) are seen over the Arabian Sea (AS) and causes the mean wave heights ranging from 2.5 to 4 m over AS (Fig. 1b). The MWP ranging from 8 to 10 s is noticed over AS, however, these periods are less when compared to rest of the TIO (10–11 s), indicated wind sea (Fig. 1d) dominance over the region (Fig. 1c). Previous studies also show that there exists a strong correlation between surface wind and wave condition over AS during boreal summer months because these regions are sheltered from distant swells and are dominated by wind waves locally^39, 40^. During JJA, AS experience a higher wind speed related to BoB due to the strong cross-equatorial winds. In BoB, mean wave heights are in the range 2–3 m. Most of the IO regions experiences strong wind fields (8–10 m/s) except equatorial regions of central and eastern IO (Fig. 1a). Tropical Southern IO (TSIO) mean wave heights are in the range (3–4 m). The wave period pattern in the TSIO indicates the swell propagation to NIO (Fig. 1c–e). During the boreal summer months, TIO surface waves display profound variability and stronger magnitudes and is mostly caused by the sub-seasonal and/or intra-seasonal variations present in the winds. Therefore, we examined the BSISO impact on TIO ocean surface waves which is potential for subseasonal predictive capability of ocean surface waves, to benefit coastal management and the analysis is given below. Understanding the relationship between BSISO (phases) and Indian Ocean wave heights also can benefit maritime operations and navigation.

**Figure 2:**
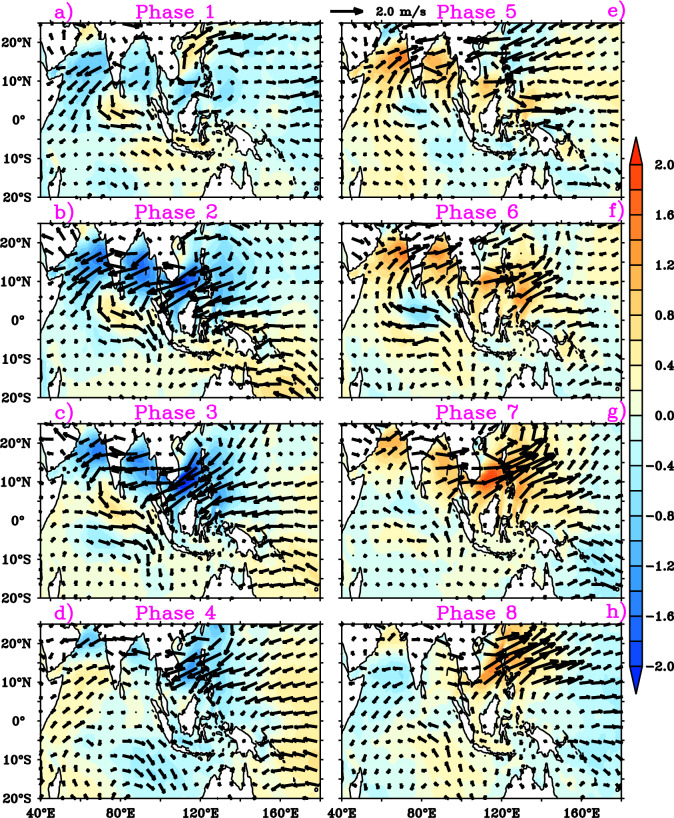
Composites of surface wind anomalies (shaded and vector; m/s) for (**a**)–(**h**) 8 BSISO phases. This figure is generated using PyFerret software (https://ferret.pmel.noaa.gov/Ferret).

The composites of surface wind anomalies for the different BSISO phases (phase 1 to phase 8) during JJA are illustrated in Fig. 2. We initially analyzed the wind speed anomalies for the eight phases of BSISO for a better understanding of wave response to BSISO. The prominent northeasterly wind anomalies over NIO and Indian subcontinent region are seen in phase 1 of BSISO (Fig. 2a). On the contrary to this, the southern tip of India region displays noticeable northwesterlies during phase 1. During phase 2 and phase 3, also easterly wind anomalies are predominant over NIO and Indian peninsula region but with stronger anomalies compared to phase 1 (Fig. 2a–c). The surface wind anomalies in phase 1–3 show a northward propagation over the NIO with weaker eastward propagation from the AS to the northwest Pacific. Phase 4 shows a reversal of wind anomalies (turned northeasterly to southwesterly) in the southwestern AS and weakening of northeasterly wind anomalies over NIO (Fig. 2d). The southwesterly wind anomalies developed in the southwestern AS in phase 4 gets strengthened and propagate northeastward over to northwest Pacific during phase 5–7 (Fig. 2e–g). In phase 7, northwest Pacific region displays strong southwesterly wind anomalies ~ 2 m/s and it also indicates the eastward propagation of wind anomalies as a response to BSISO. Phase 8 shows reversal of wind anomalies in southwestern AS (turned southwesterlies to northeasterlies) and weakening of southwesterly wind anomalies over NIO (Fig. 2h). Over the southern tip of India and in the equatorial central Indian Ocean (60°E–80°E), westerly (easterly) wind anomalies prevail during phase 2 and phase 3 periods (phase 5 to phase 7). In short, the southwesterly anomalies develop in the southwestern AS in phase 4 shifts into the BOB and maritime continents in phase 5, and propagate over the northwestern Pacific Ocean through phases 6–8. Meanwhile, northeasterly wind anomalies build up over the southwestern AS in phases 8 propagate over the Maritime Continent and BOB in phase 1 and extend across the northwestern central Pacific in phases 2 and 3. BSISO phases 1–3 shows suppressed winds over AS, BOB, western TSIO and western Pacific (Fig. 2a–c), whereas phases 5–7 feature strong westerlies over the said regions (Fig. 2e–g). Phases 4 and 8 appear as transition phases for these regions.

**Figure 3:**
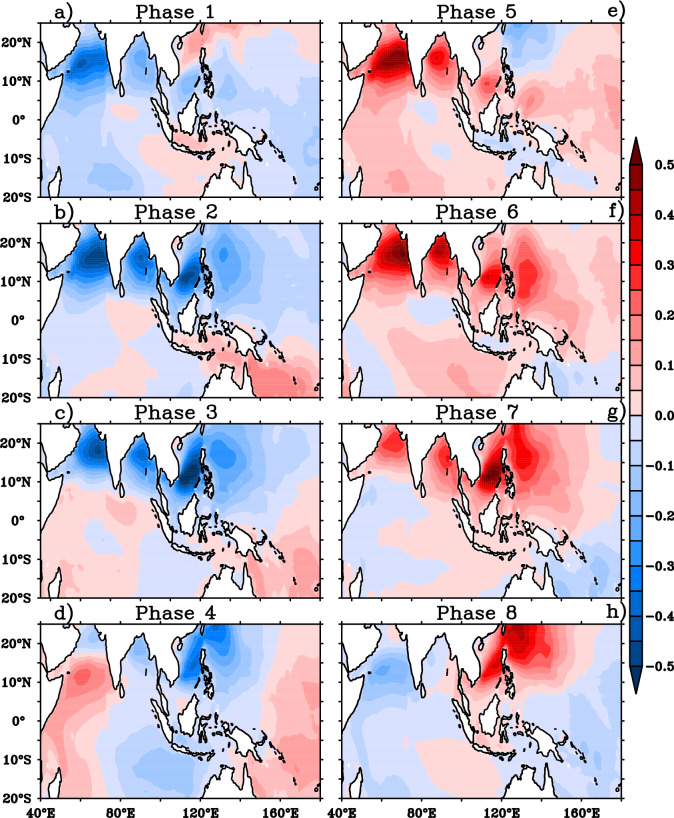
Composites of significant wave height anomalies (shaded; m) for (**a**)–(**h**) BSISO 8 phases. This figure is generated using PyFerret software (https://ferret.pmel.noaa.gov/Ferret).

**Figure 4:**
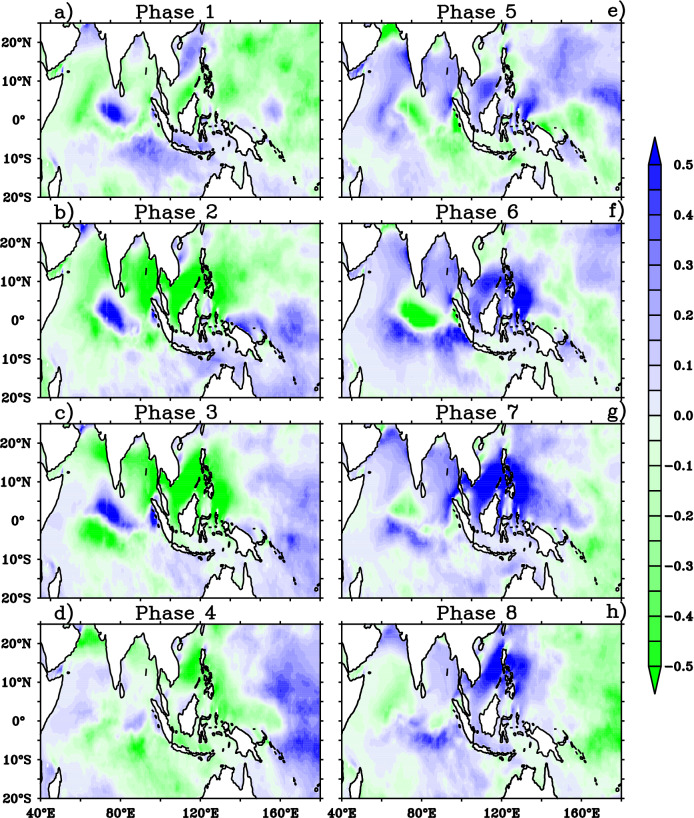
Composite anomalies of normalized energy flux into waves (shaded; Nm^2 ^s^−1^) for (**a**)–(**h**) BSISO 8 phases. This figure is generated using PyFerret software (https://ferret.pmel.noaa.gov/Ferret).

The response of wave heights to the BSISO induced wind anomalies for the Indo-western Pacific Ocean is explored using the composites of SWH anomalies for different BSISO phases (Fig. 3). The strong negative SWH anomalies over western AS and moderate negative anomalies over BoB are noticed during the phases 1 to 3. The significant negative SWH anomalies with strong northward and weak eastward propagation from the western AS to northwest Pacific are identified during phases 2 and 3 as a response to BSISO. During phase 1–3, northeasterly wind anomalies were acting on mean southwesterly winds which caused the significant reduction of wave growth by ~ 50 cm over the Indo-western Pacific region (Figs. 1a, 2a–c). The composites of anomalies of normalized energy flux transfer from the wind to waves are displayed in Fig. 4 and the reduced energy flux into waves further supports the reduction (increase) of wave heights over NIO during the phases 2–3 (phase 5–7) (Fig. 4b–c, e–g). Most of the northern Indo-western Pacific region SWH anomalies are negative (~ 50 cm) with negative anomalies of energy flux transfer into waves from the winds where BSISO northeasterly anomalies act on mean state southwesterly winds during phases 1–3. Conversely, SWH anomalies are positive (~ 50 cm) with positive anomalies of energy flux transfer to waves where BSISO southwesterly anomalies act on mean state southwesterly winds during phases 5–7. Therefore, it is important to point out that the intraseasonal variability in the wave heights is highly modulated by the energy transfer into waves from the winds during JJA in response to the BSISO mode.

The reduced energy flux into waves further supports the reduction (increase) of wave heights over NIO during the phases 2–3 (phase 5–7) (Fig. 4b–c, e–g). Most of the northern Indo-western Pacific region SWH anomalies are negative (~ 50 cm) with negative anomalies of energy flux transfer into waves from the winds where BSISO northeasterly anomalies act on mean state southwesterly winds during phases 1–3. Conversely, SWH anomalies are positive (~ 50 cm) with positive anomalies of energy flux transfer to waves where BSISO southwesterly anomalies act on mean state southwesterly winds during phases 5–7. Therefore, it is important to point out that the intra-seasonal variability in the wave heights is highly modulated by the energy transfer into waves from the winds during JJA in response to the BSISO mode.

**Figure 5:**
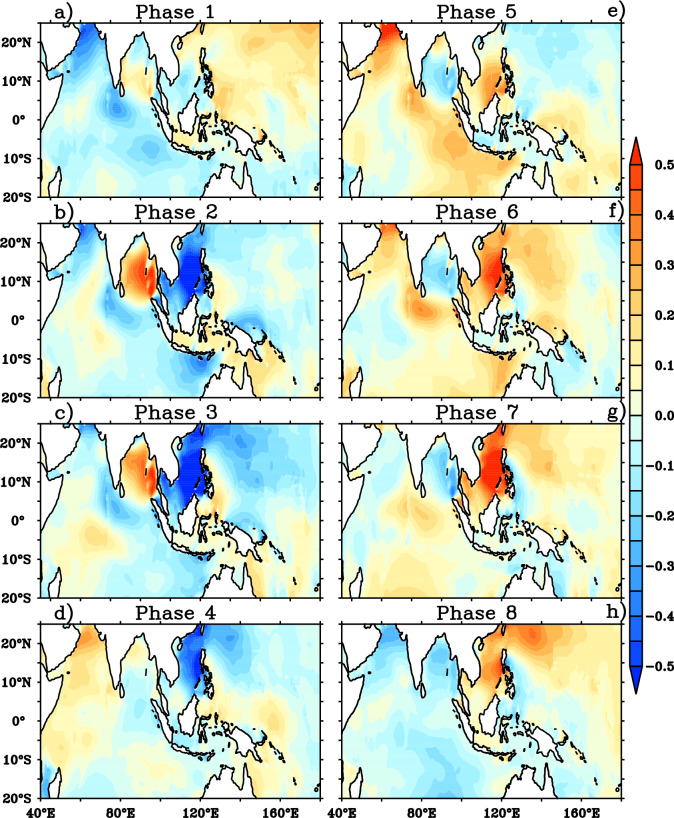
Composites of mean wave period anomalies (shaded; s) for (**a**)–(**h**) BSISO 8 phases. This figure is generated using PyFerret software (https://ferret.pmel.noaa.gov/Ferret).

**Figure 6:**
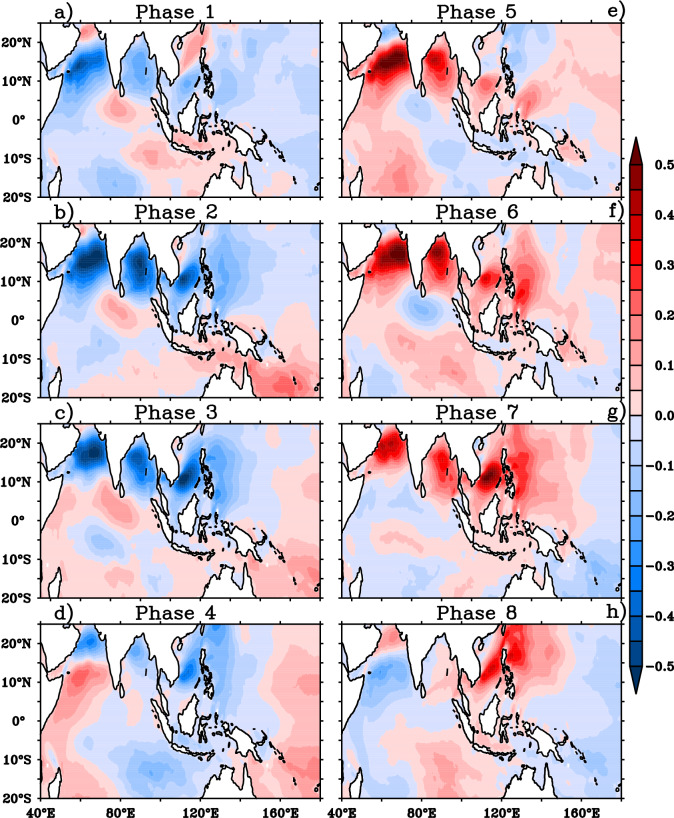
Composite anomalies of significant height of wind waves (shaded; m) for (**a**)–(**h**) BSISO 8 phases. This figure is generated using PyFerret software (https://ferret.pmel.noaa.gov/Ferret).

**Figure 7:**
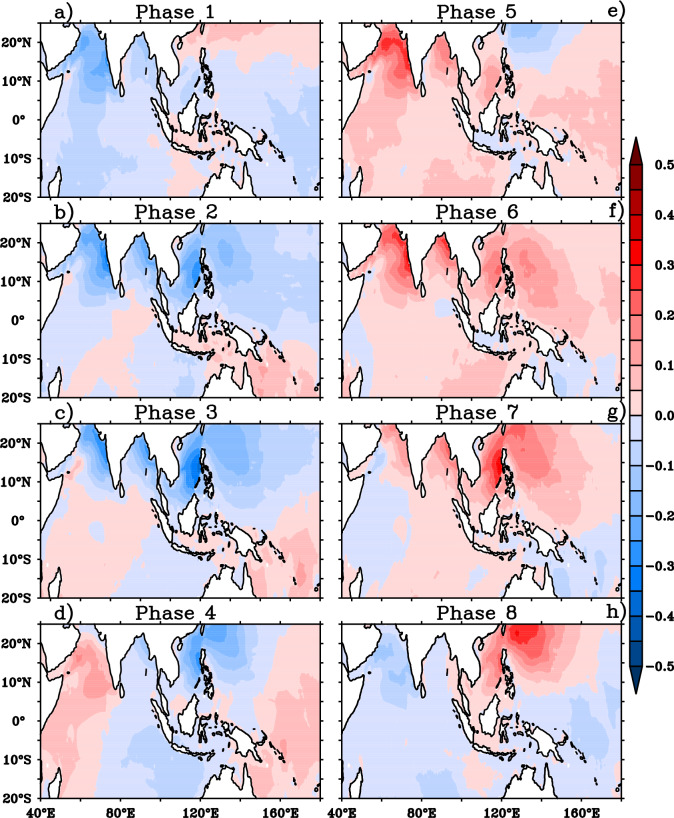
Composite anomalies of significant height of total swell (shaded; m) for (**a**)–(h) BSISO 8 phases. This figure is generated using PyFerret software (https://ferret.pmel.noaa.gov/Ferret).

The impact of BSISO on mean wave heights can be further explained with the help of the composite of MWP anomalies which are illustrated in Fig. 5. The negative MWP anomalies over AS, northern BOB and positive anomalies over southern BOB are clearly visible during phases 1–3 (Fig. 5a–c). The positive MWP anomalies over southern BOB further intensified with northward extension and occupied the entire BOB during the BSISO phases 2 and 3. The southern tip of India and northwest Pacific Ocean including the maritime continent displays negative MWP anomalies during phases 2–3. It is noticed that the negative MWP anomalies over AS are replaced with positive anomalies during phase 4–6 as a response to BSISO and it is much consistent with SWH anomalies over AS (Fig. 5d–f). Similarly, the northwest Pacific also displays positive MWP anomalies during these phases. Interestingly, the wave period anomalies shows an out-of phase relation with SWH anomalies in BOB and an in-phase variation with SWH anomalies in AS during the entire cycle (phases 1–8). This could be attributed by the sea and swell analysis. Therefore, the composites of significant height of wind waves (Hsw) and significant height of total swell (Hss) for different BSISO phases is also analyzed (Figs. 6, 7).

During JJA, the AS is dominated by mixed sea state i. e, a combination of strong monsoon winds forced wind sea and swell^22,29^ and peak wave period above 15 s is rarely seen in this period indicating non-dominance of SIO swells. A recent study shows that the SIO swell propagation is more towards BOB during JJA^41^. These points set the background for the explanation of in phase relation of MWP anomalies with SWH in AS. A decrease/increase in the local wind will result in a reduction/increase of energies in the high frequencies. In AS, a reduction/increase in the monsoon wind reduces/increases both wind sea and swell (Figs. 6, 7). Negative anomalies of Hss and Hsw is seen during phase 1–3 (characterized by negative anomalies of winds) in the AS (Figs. 6a–c, 7a–c) indicating the reduction in both. During phases 5–7 (characterized by positive anomalies of winds), positive anomalies of both Hss and Hsw is seen in the AS indicating the active sea generation and nonlinear energy transfer to swell in the area^42,43^ (Figs. 2, 4, 6, 7e–g). The variations of northwestern Pacific wave parameters also similar to that of AS indicate the wave fields are mostly controlled by the local winds. But the BOB MWP anomalies showed an out of phase variation with Hs anomalies in all the 8 phases of BSISO. BOB is an SIO swell dominated region and hence a decrease in local winds will result in less energy in the higher frequencies and cause the shift of peak frequency to the lower frequencies and thus, on average, increasing MWP. This can be seen in BOB wave anomalies during the phases 1–3 (Fig. 5a–c). Conversely, BSISO phases 5–7 characterized by positive anomalies of winds will result in an increase of energy in the higher frequencies and cause a decrease in the MWP as seen in Fig. 4e–g. Positive anomalies of Hsw and Hss seen during these phases are due to the increase of monsoon wind generated sea and swell and support the above fact (Fig. 5a–c). In short, BSISO phases have a significant impact on the wave characteristics of NIO during JJA.

**Figure 8:**
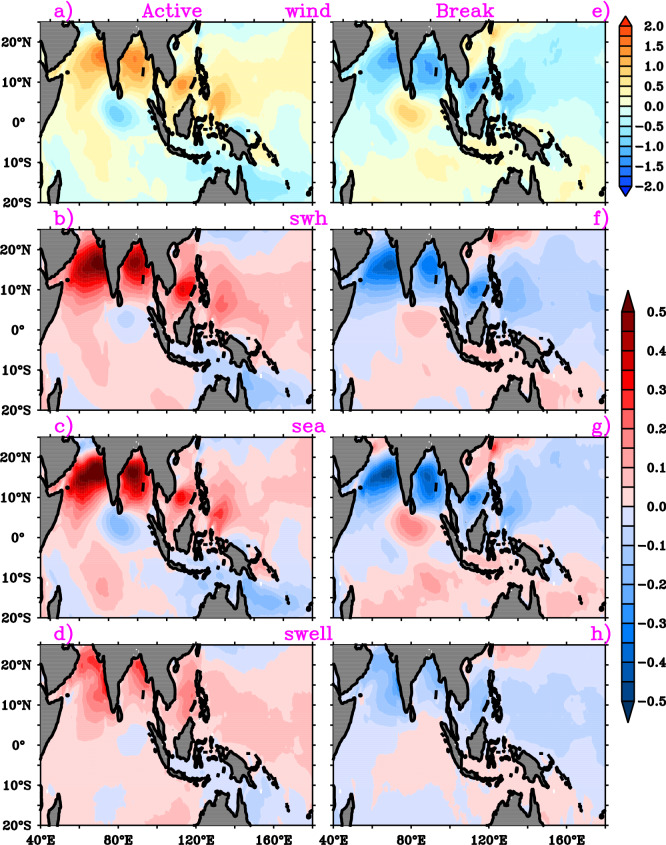
Composite anomalies of (**a**) surface winds, (**b**) significant wave height, (**c**) significant height of wind sea and (**d**) significant height of total swell during the active phase of BSISO. (**e**)–(**h**) are same as (**a**)–(**d**) during break phase of BSISO. This figure is generated using PyFerret software (https://ferret.pmel.noaa.gov/Ferret).

The active and break cycles of intra-seasonal oscillations influences the Indian summer monsoon seasonal mean rainfall and circulation^9,43–45^. Thus, we have analyzed the wave conditions during the active and break spells of BSISO (Fig. 8). Enhanced (suppressed) wave height anomalies (~ 0.5 m) are noticed during the active (break) spells in response to strong (weak) southwesterly wind anomalies over NIO. Over the southern tip of India, negative (positive) wave height anomalies prevail during the active (break) conditions. The active phase SWH anomaly spatial pattern is quite similar to the seasonal mean wave pattern and can play a role in strengthening the seasonal mean (Fig. 8a,b). In addition, the composites of the sea and swell anomalies show that the strong (weak) SWH anomalies in the active (break) phase are contributed by the wind wave anomalies. The swells impact is less comparatively as the NIO is mostly dominated by the monsoon driven southwesterly winds during JJA. The study reveals that the intra-seasonal oscillation during JJA modulates the TIO ocean surface waves. Prediction of these oscillations in the extended and short-range forecast for the Indian Ocean rim countries will be beneficial for various marine applications.

## Summary and conclusion

In the present study, we have analyzed the influence of boreal summer intra-seasonal oscillation (BSISO) on ocean surface waves during the boreal summer monsoon (JJA) over the Indo-western Pacific Ocean using the ERA5 reanalysis covering the period 1979–2017. BSISO is one of the major sources of short-term climate variability during the summer monsoon and it has a potential influence on the seasonal mean modulation^6,16,46^. However, the impact of BSISO on surface waves which is crucial for coastal and offshore management is not addressed previously. The composite analysis is carried out on the different phases of BSISO. The southwesterly wind anomalies develop in the southwestern AS in phase 4, shift into the BOB and maritime continents in phase 5, and propagate over the northwestern Pacific through phases 6–8. Meanwhile, northeasterly wind anomalies build up over the southwestern AS in phases 8 propagate over the Maritime Continent and BOB in phase 1 and extend across the northwestern central Pacific in phases 2 and 3. SWH anomalies in response to BSISO’s are phase-dependent. The composites of SWH anomalies displayed negative anomalies with strong northward and weak eastward propagation during the phases 1–3 in response to the easterly wind anomalies that act on the mean westerly anomalies over NIO. During phase 5–7, the SWH anomalies displayed high positive anomalies (~ 0.5 m) in response to the westerly wind anomalies that act on mean westerlies with northward and weak eastward propagation over NIO.BSISO phases 1–3 show suppressed winds and waves over AS, BOB, western TSIO, and western Pacific, whereas phases 5–7 feature strong westerlies and positive SWH anomalies over the regions. Phases 4 and 8 appear as transition phases for these regions. The composite anomalies of MWP, Hsw and Hss confirmed the SWH anomalies are caused by the increase in the wind sea which in turn connected to the BSISO induced strong southwesterly winds during phases 5–7. The development of northeasterly wind anomalies over the southwestern AS in phase 8 decreases the local wave growth. The negative SWH anomalies extend over the Maritime Continent and BOB in phase 1 and cover the northwestern central Pacific, in phases 2 and 3 with the propagation of wind anomalies. The composites of normalized energy flux transfer from wind to wave further support the discussion of negative SWH anomalies with negative flux anomalies during the phases 1–3 and positive SWH anomalies with positive flux anomalies during the phase 5–7 over NIO.

In addition, the active phases of BSISO create significant positive SWH anomaly over NIO during monsoon and the high SWH anomalies are seen over the AS (~ 0.5 m). During the active phase of BSISO, AS experiences high wave activity and this may have an adverse impact on marine activities especially for navigation. Usually, the west coast of India experiences coastal flooding and severe erosion during monsoon due to high waves approaching the coast. In the active phase of BSISO, the west coast of India experiences intense erosion and more floods. Conversely, the break phase of BSISO reduces the wave activity over the AS. This could be helpful for offshore and coastal management. Our analysis reveals that the BSISO mode has a significant influence on the ocean surface wave variability over the Indo-western Pacific Ocean. The wave forecast advisories based on the BSISO would be more useful for efficient coastal and marine management as the monsoon induced surface waves to have a large impact on marine activities. Further research needs to be carried out for a better understanding of the impact of BSISO induced waves on coastal erosion and other coastal processes.
